# Pediatric Influenza Prevention and Control

**DOI:** 10.3201/eid1004.030398

**Published:** 2004-04

**Authors:** Nicola Principi, Susanna Esposito

**Affiliations:** * Institute of Pediatrics, University of Milan, Milan, Italy

**Keywords:** Influenza, vaccine, prevention, children, socioeconomic problems

## Abstract

Global evaluation of influenza vaccination in children indicates that current recommendations are not followed. Most children at high risk for influenza-related complications do not receive the vaccine, and increased efforts are needed to protect them. Furthermore, immunizing healthy infants 6–23 months of age and their close contacts should be strongly encouraged. Vaccinations are recommended for children with recurrent acute otitis media or recurrent respiratory tract infections and possibly for healthy daycare and school-age children because of the potential socioeconomic implications of influenza. Issues that need to be addressed include educating physicians and parents concerning influenza-related illness and complications, cost-effectiveness and safety of licensed vaccines, adequate vaccine supply, and availability of intranasal products.

Influenza vaccination is routinely recommended in pediatric patients of age >6 months who are at high risk for influenza-related complications because they have an underlying disease or are undergoing long-term aspirin therapy and are at risk of developing Reye syndrome (*1*–*4*). Administering the vaccine to healthy children is recommended only when they live with persons at high risk (*1*–*4*), although the Advisory Committee on Immunization Practices is gradually moving toward a recommendation to vaccinate all children ages 6–23 months (because of their substantially increased risk for influenza-related hospitalizations) and children ages 2–18 years who are household contacts of children ages 0–23 months (*1*–*7*).

Although health authorities in industrialized countries agree with these guidelines, use of influenza vaccine in clinical practice differs. Most children at high risk for complications do not receive the vaccine, and its use in healthy infants is not routinely accepted (*8*–*10*), even though results of recent studies suggest expanding the number of children for whom vaccination should be recommended (*11*–*14*). We discuss current vaccination practices in children, reasons and possible remedies for low immunization rates, and the possibility of extending its use in pediatrics.

## Vaccine Practices for Children

High-risk children for whom influenza vaccination is routinely recommended include those with chronic disorders of the cardiovascular or pulmonary system (including asthma), chronic metabolic diseases (including diabetes mellitus), chronic renal dysfunction, and hemoglobinopathies or immunosuppression (including cases caused by medications or by HIV) (*1*–*4*). Although an association between these conditions and an increased risk for influenza complications was first suggested many years ago (*15*,*16*), the level of vaccination in such children is still much lower than recommended, although it is slightly higher when children are followed up in specialized centers rather than by primary care physicians (perhaps because children seen in specialty clinics have more severe underlying illnesses), or when data regarding immunization are collected after implementing a reminder and recall system (*8*–*10*,*17*–*19*). One study of health maintenance organizations reported influenza vaccination rates of 9% to 10% among children with asthma and a rate of 25% among those attending an allergy and immunology clinic (*17*). The use of a reminder and recall system increased vaccination coverage among children with asthma from 5% to 32% (*18*). The highest coverage was found among pediatric patients attending a cystic fibrosis treatment center, in whom a vaccination level of 79% was reached (*19*). Data collected in Italy confirm that the behavior of pediatricians is not in line with the official recommendations. Among the 274 high-risk children attending the University of Milan’s Pediatric Emergency Department during winter 2002–2003, the vaccination level was 26.3%; the highest rates were in children with HIV infection (52.3%), and the lowest rates were in those with asthma (9.5%) (*10*).

Few data concern the effect of encouraging vaccination in healthy children <2 years. However, comparing immunization rates among children of this age without any high-risk condition attending the University of Milan’s Pediatric Emergency Department during the two winter seasons of 2001 to 2002 and 2002 to 2003 (after the publication of the suggestion that healthy children <2 years be vaccinated) showed only a marginal increase (2.4% vs. 3.6%) (*10*).

## Reasons for Low Immunization Rates and Possible Solutions

Seven main obstacles to complying with recommendations for vaccination in children exist: 1) lack of understanding of the risk for influenza complications in children; 2) lack of knowledge of annual immunization’s efficacy in primary prevention; 3) parents’ negative reaction to parenteral vaccine administration (“Not another shot!”); 4) need for two priming doses in children <9 years old followed by annual administration; 5) fear of limited protection in younger and high-risk children; *6*) concerns about possible adverse events; and 7) lack of precision in current recommendations. The most important of these obstacles are lack of understanding of the risks for complications and lack of knowledge of efficacy (*10*,*20*).

A number of studies of adult (particularly elderly) populations have shown that knowing risk factors for influenza complications, favorable perceptions of the vaccine, and clinician recommendations are the main variables predicting the administration of influenza vaccination (*1*,*21*,*22*). However, pediatric data indicate that some providers do not recognize influenza’s clinical relevance, even when it occurs in children with severe underlying disease (*8*,*9*). A study designed to ascertain the self-reported use of influenza vaccine among pediatric oncologists found that approximately 30% did not think that influenza infection is important in children with cancer (*8*) and consequently do not recommend immunization. The central role of physicians’ opinions in determining vaccination coverage is supported by data collected in a cross-sectional study of a group of children hospitalized during the influenza season in the United States (*9*): >70% of the children were vaccinated if a physician had recommended it to their parents, but 3% were vaccinated if no such recommendation had been made. A lack of awareness that children can receive influenza vaccine was a commonly cited reason for nonvaccination (*9*).

The attitude of pediatricians towards influenza vaccine can be explained by the fact that its importance in high-risk children and healthy infants is mainly suggested by indirect data. Although a number of studies have shown that influenza can significantly increase hospitalization, outpatient visits, and drug consumption in high-risk children of all ages (*15*,*16*), few trials (mainly involving children with asthma) have demonstrated that vaccination is clinically useful in reducing influenza-related complications (*23*,*24*). Furthermore, data concerning the efficacy of influenza vaccine in healthy infants <2 years of age have been collected from small groups. Although a reduction in influenzalike illnesses has been shown, the data do not evaluate the importance of vaccination in reducing hospitalizations or complications (*25*,*26*). Pediatricians may be definitively convinced of the importance of preventing influenza and personally start supporting the use of vaccine when more data are available demonstrating its efficacy in children. Consequently, studies evaluating the real clinical impact of influenza vaccine, not only in children with risk factors but also in healthy infants, are needed.

Another probable factor preventing the use of influenza vaccines in pediatrics is that those currently licensed for use in children are parenteral (two injections for children <9 years of age being vaccinated for the first time) and require annual administration to maintain protection (*1*,*3*,*27*). Parents may be concerned about the number of injections their children receive during the course of routine early child health visits. Given the large number of vaccinations already included in the routine childhood immunization schedule, the addition of another “shot” may not sound attractive to parents and certainly not to their children. However, the availability of intranasal influenza vaccines may substantially reduce this problem (*28*). Recent advances in influenza vaccination include the development of a trivalent, cold-adapted, live-attenuated, intranasal vaccine that appears to be as effective as its intramuscular counterparts and induces a good immune response (including local immunoglobulin [Ig] A responses and secretory IgA antibodies that can protect against pathogens infecting mucosal sites) (*29*). One of the disadvantages of this vaccine is that individual susceptibility to infection with live viruses (and consequent immunogenicity) varies widely; vaccine strains’ reversion to their wild-type genotype has also been considered a potential risk, although there is no evidence that this occurs (*29*). If eventually licensed for use worldwide, intranasal vaccines can be expected to increase influenza vaccination coverage, especially in children.

Concerns that influenza vaccine may offer limited protection and fears of possible adverse events are further reasons for its limited use in pediatrics (*20*). However, protective antibody levels after influenza vaccination have developed in 70% to 90% of children as young as 6 months of age, although fewer younger infants seroconvert, and some high-risk children may have a lower antibody response (*1*). Childhood vaccination programs fail to be beneficial if vaccine efficacy falls to <25%, levels that have never been reported in younger or high-risk children (*1*). Moreover, although mild local and systemic reactions to the vaccine may occur more frequently in persons who have never been exposed to the viral antigens it contains (e.g., young children), the currently licensed parenteral vaccines are generally safe and well-tolerated (*1*). Considering the possible effect that “vaccine-adverse” parents have on immunization policy in some regions, disseminating information concerning the safety, tolerability, and immunogenicity of influenza vaccination in healthy infants and high-risk children is important.

Influenza prevention recommendations imprecisely describe the characteristics of high-risk children, contributing to inadequate vaccination in this population. For example, the Advisory Committee on Immunization Practices recommends yearly influenza vaccination for immunosuppressed children, including those with immunosuppression due to medications (*1*) but does not specify which diseases require vaccination, the doses of the immunosuppressive drugs, or the timing of the vaccination in relation to their administration (*1*). Conversely, the American Academy of Pediatrics states that the optimal time to immunize these children is when their peripheral leukocyte count is >1,000/μL and that vaccination has to be deferred during high-dose corticosteroid administration (*27*). These discrepancies reflect a lack of data and may explain why pediatricians have different approaches in clinical practice. Specific and uniform guidelines for each group of children at high risk would be the best way to overcome this problem. Still, in many clinical scenarios decisions are based on the best information available, and recommendations cannot deal with each and every situation that the medical provider confronts.

Globally evaluating the main reasons for low influenza vaccination coverage in pediatrics suggests that improving knowledge of influenza among pediatricians and parents could improve vaccination practices. The medical community spends substantial amounts of time with parents trying to convince them of the need for routine vaccinations, but in many instances, vaccines are suggested on the basis of the parents’ or the healthcare providers’ perception of vaccine or diseases of greatest importance. If parents lack insurance, economic considerations also become an issue. A change of mindset is needed to enhance acceptance of influenza vaccination; providing materials to educate parents would help effect this change. As television and print advertising promotes other pharmaceutical products, similar advertising could effectively promote influenza vaccination. The first step is to define simple, unequivocal, and practical guidelines specific to different groups of children at high risk and healthy infants <2 years of age. These guidelines, for distribution to hospital physicians and primary care pediatricians, would contain detailed information regarding the consequences of influenza in such children and describe the effectiveness of influenza vaccine and the risk for adverse events. In addition, pediatricians can use recall systems to provide timely reminders for all patients.

## Influenza Vaccine in Children Not at Risk

In addition to the children for whom influenza vaccine is already recommended or strongly encouraged, other pediatric patients can receive clinical benefits from its use. One group of children who could be included on the list of vaccination candidates is those with recurrent episodes of acute otitis media (AOM). Recurrent AOM is common in infants and children, and its possible sequelae make prevention desirable (*30*). Until a few years ago, chemoprophylaxis and controlling environmental risk factors were considered the best ways to reduce the incidence of new episodes of AOM in otitis-prone children, but the emergence of drug-resistant bacteria after antimicrobial drug administration raises questions about the advisability of drug therapy (*13*,*30*). Immunoprophylaxis against respiratory viruses has received growing attention because viral infections (including influenza) are associated with many, if not most, episodes of AOM. Data showing that administering parenteral, inactivated influenza vaccine can decrease the incidence of AOM by approximately one third strongly support the use of vaccination in preventing AOM (*31*). The demonstration that live-attenuated, cold-adapted, intranasal vaccine causes a 30% reduction in the incidence of febrile AOM in healthy children without a history of ear disease leads to the same conclusion (*25*). The importance of influenza vaccination in children with recurrent AOM has been recently demonstrated by Marchisio et al., who used an intranasal, inactivated, virosomal subunit vaccine (*13*). In this study, 133 children aged 1–5 years with recurrent AOM (defined as >3 episodes in the preceding 6 months or >4 episodes in the preceding 12 months) were randomized to receive the vaccine (n = 67) or no vaccination (n = 66). During a 6-month period, 24 vaccine recipients (35.8%) experienced 32 episodes of AOM, and 42 control participants (63.6%) experienced 64 episodes. The overall efficacy of vaccination in preventing AOM was 43.7% (95% confidence interval 18.6 to 61.1, p = 0.002) (Table 1) (*13*). Moreover, the cumulative duration of middle ear effusion was significantly less in the vaccinated children (58.0% vs. 74.5%; p < 0.0001) (*13*). As reducing the occurrence of AOM in children with recurrent episodes can have substantial clinical and socioeconomic effects, these data suggest that influenza vaccine can be considered a valid option in preventing the disease in otitis-prone children.

**Table 1:** Effectiveness of influenza vaccine as indicated by the occurrence of febrile respiratory illness and acute otitis media (AOM), and the use of antibiotics in children during the 6 months after vaccine administration^a^

Variable	Vaccine recipients, n = 67 (%)	Control participants n = 66, (%)	Vaccine efficacy, %	p value
Febrile respiratory illness	55 (82.1)	63 (95.5)	13.2	0.03
>1 course of antibiotics	26 (38.8)	42 (63.6)	38.9	0.007
>1 AOM episode	24 (35.8)	42 (63.6)	43.7	0.002
>2 AOM episodes	6 (9.0)	16 (24.2)	63.1	0.03

^a^Modified from P. Marchisio et al. (*13*).

A second group of children who could be considered for influenza vaccine are those with recurrent episodes of respiratory tract infections (RRTIs). A large number of children without any immunologic problems experience multiple episodes of RRTIs during the first years of life; although these generally have a benign prognosis, they can cause substantial medical and socioeconomic problems (*32*). They are mainly caused by viruses and, during epidemic periods, influenza viruses can also be causative. Data collected in a recent study indicate that vaccinating children with RRTIs against influenza is effective in decreasing respiratory-related illness among them and their families (*14*). A total of 127 children 6 months to 9 years of age with a history of RRTIs (>6 episodes per year if >3 years; >8 episodes per year if <3 years) were randomized to receive the intranasal virosomal influenza vaccine (n = 64 with 176 household contacts) or a control placebo (n = 63 with 173 household contacts). During the influenza season, vaccinated children had fewer respiratory infections or febrile respiratory illnesses, received fewer prescribed antimicrobial and antipyretic drugs, and missed fewer school days than the controls (Table 2); similar benefits and a reduced loss of parental work were observed among their household contacts (*14*). These results show that the benefits of influenza vaccination extend to children with RRTIs and their families and suggest that its use in such children should be encouraged.

**Table 2:** Respiratory illness among children with recurrent respiratory tract infections and effectiveness of the influenza vaccine during the follow-up period^a^

Event	Vaccinated children, n = 64)^b^	Controls, n = 63^b^	Vaccine effectiveness, %^c^	p value
No. of upper respiratory tract infections	2.95 ± 1.33 (3)	4.06 ± 2.13 (4)	27	<0.0001
No. of lower respiratory tract infections	0.67 ± 0.88 (0)	1.01 ± 1.12 (1)	33	0.03
No. of febrile respiratory illnesses	1.60 ± 1.39 (1)	2.06 ± 2.14 (2)	23	0.02
No. of hospitalizations	0.05 ± 0.10 (0)	0.10 ± 0.25 (0)	60	0.34
No. of antimicrobial prescriptions	1.31 ± 1.33 (1)	2.35 ± 1.59 (2)	44	<0.0001
No. of antipyretic prescriptions	2.16 ± 2.03 (2)	3.98 ± 2.37 (4)	45	<0.0001
Missed school days	5.35 ± 8.14 (6)	13.83 ±12.50 (10)	61	<0.0001

^a^Modified from S. Esposito et al. (*14*). ^b^Mean values ± standard deviation (median in parentheses). ^c^Vaccine effectiveness: 1 minus attack rate (defined as rate of illness divided by total population) among vaccinated children divided by attack rate among controls.

## Influenza Vaccine in Healthy Daycare and School-Aged Children

A number of studies have shown that otherwise healthy daycare and school-aged children are most frequently affected by influenza, and high attack rates can substantially diminish their quality of life and disrupt everyday activities (*33*–*36*). Children shed larger quantities of influenza viruses for longer periods of time than adults and thus play an important role in spreading infection in their families and communities (*1*,*37*). Negative effects of influenza in otherwise-healthy children can extend to unvaccinated household contacts, who may require substantial diagnostic and therapeutic interventions and miss a number of school or working days. Neuzil et al. found that, during the influenza season, the number of household members who became ill within 3 days of a child’s absence from school was 2.2 times higher than expected. Excess absenteeism from work also occurred among parents (*34*). In line with these observations, we have found that the household contacts of children with influenza require more medical visits, miss more working or school days, and need more help at home to care for ill children than the household contacts of children without influenza (*36*).

Preventing influenza by vaccination can improve these situations. A blinded, placebo-controlled study of two influenza vaccines (an inactivated split-virus vaccine and a live-attenuated, cold-adapted vaccine) in 555 school-aged children in Russia demonstrated that both were efficacious in preventing school absenteeism by reducing the number of missed school days by 47% to 56% compared to missed school days in unvaccinated children (*38*). Similarly, in a study of the effect of an inactivated, split-virus vaccine on healthy children attending daycare or school in Italy during the years 2001–2002, we found that the vaccinated children experienced fewer upper and lower respiratory tract infections, received fewer antimicrobial and antipyretic prescriptions, and missed fewer school days because of respiratory illnesses (*39*). These data suggest that the effect of influenza on otherwise-healthy daycare or school-age children may be more substantial than is usually thought, encouraging wider pediatric use of influenza vaccine to reduce the overall extent of infection.

Strong support for wider pediatric use comes from evaluating the household impact of influenza vaccination in healthy daycare and school-age children. In a 1995 randomized, controlled trial of influenza vaccine for preschool children, the rate of febrile respiratory illnesses was 42% less among the unvaccinated household contacts of influenza-vaccinated children than among those living with unvaccinated children (*40*). Moreover, data collected in Tecumseh, Michigan (*41*), and Japan (*7*) indicate that mass vaccination of school-age children correlates with a reduced rate of respiratory illness and all-cause community death rate, which suggests that larger scale immunization can affect community epidemics. Similarly, during the 2001–2002 influenza season in Italy, we found that, compared to the household contacts of unvaccinated children, family members of influenza-vaccinated healthy children experienced fewer respiratory tract infections, needed fewer medical visits, missed fewer working days, and required less help at home to care for ill children (Table 3) (*39*). All of these findings highlight the fact that influenza in otherwise-healthy children attending daycare centers or schools has a considerable effect on their families and that the benefits of influenza vaccination extend to the family members of vaccinated persons.

**Table 3:** Effectiveness of influenza vaccine among household contacts of children receiving influenza vaccine and unvaccinated controls^a^

Event	Household contacts of vaccinated children (n = 728)^b^	Household contacts of unvaccinated controls (n = 370)^b^	Vaccine effectiveness, %^c^	p value
No. of respiratory tract infections	3.03 ± 1.68	4.27 ± 1.68	30	0.0005
No. of medical visits because of respiratory illness	2.18 ± 1.37	3.16 ± 1.77	32	0.002
Loss of maternal work, days	3.22 ± 1.86	4.78 ± 2.34	33	0.001
Loss of paternal work, days	0.56 ± 0.46	0.98 ± 2.24	43	0.001
Help at home to care for ill children, days	0.57 ± 0.37	3.22 ± 2.24	83	<0.0001

^a^Modified from S. Esposito et al. (*39*). ^b^Mean values ± standard deviation. ^c^Vaccine effectiveness: 1 minus attack rate (defined as rate of illness divided by the total population) among household contacts of vaccinated children divided by attack rate among household contacts of controls.

The socioeconomic importance of influenza in childhood is confirmed by economic analyses showing that vaccinating healthy preschool and school-age children can lead to health and economic benefits during epidemic and pandemic periods (*42*–*45*). These studies used different analytic methods, outcomes, and costs but came to a common conclusion: vaccinating healthy children against influenza leads to a net cost saving, and the greatest financial benefit is observed when the vaccine is administered in a group setting (*42*–*45*). Savings are primarily due to avoided indirect costs and, in particular, reduced parental absenteeism from work.

## Conclusion

Global evaluation of the effect of influenza in pediatric patients indicates that influenza vaccination should be more widely used than is usually recommended. To protect them against the complications of influenza, increased efforts are needed to identify and recall high-risk children. Further, immunizing infants 6–23 months of age and their close contacts is recommended. Children with recurrent AOM or a history of RRTIs and healthy children attending daycare centers or schools should also be included among the pediatric groups recommended for vaccination.

These conclusions are based on clinical and socioeconomic considerations arising from evaluating the impact of influenza vaccination on both the children themselves and their household contacts. Improved recognition of the complications of influenza in the first years of life, with resources dedicated to provider and public education on this issue, can help reduce obstacles to using influenza vaccine. Parents might choose vaccination for their children if they were more informed about the health and economic cost of influenza, its annual attack rate in childhood (which leads to days lost from school and work), and the central role of children in disseminating the infection in households and communities. The issues that need to be addressed include educating physicians and parents about the illness caused by influenza, the cost-effectiveness and safety of licensed vaccines, adequate vaccine supplies, and the availability of intranasal products. Improved compliance associated with nasal administration should increase the use of influenza vaccination. Only a heightened and regular demand for influenza vaccine will result in sufficient vaccine supplies at all times (not just on a year-to-year basis) and place us in a better position to detect a novel pandemic influenza virus strain.
